# Similarities and Differences between Individuals Seeking Treatment for Gambling Problems vs. Alcohol and Substance Use Problems in Relation to the Progressive Model of Self-stigma

**DOI:** 10.3389/fpsyg.2017.00957

**Published:** 2017-06-09

**Authors:** Belle Gavriel-Fried, Tal Rabayov

**Affiliations:** Bob Shapell School of Social Work, Tel Aviv UniversityTel Aviv, Israel

**Keywords:** self-stigma, public stigma, problem gambling, alcohol use problems, substance use problems

## Abstract

**Aims:** People with gambling as well as substance use problems who are exposed to public stigmatization may internalize and apply it to themselves through a mechanism known as self-stigma. This study implemented the Progressive Model for Self-Stigma which consists four sequential interrelated stages: awareness, agreement, application and harm on three groups of individuals with gambling, alcohol and other substance use problems. It explored whether the two guiding assumptions of this model (each stage is precondition for the following stage which are trickle-down in nature, and correlations between proximal stages should be larger than correlations between more distant stages) would differentiate people with gambling problems from those with alcohol and other substance use problems in terms of their patterns of self-stigma and in terms of the stages in the model.

**Method:** 37 individuals with gambling problems, 60 with alcohol problems and 51 with drug problems who applied for treatment in rehabilitation centers in Israel in 2015–2016 were recruited. They completed the Self-stigma of Mental Illness Scale-Short Form which was adapted by changing the term “mental health” to gambling, alcohol or drugs, and the DSM-5-diagnostic criteria for gambling, alcohol or drug disorder.

**Results:** The assumptions of the model were broadly confirmed: a repeated measures ANCOVA revealed that in all three groups there was a difference between first two stages (aware and agree) and the latter stages (apply and harm). In addition, the gambling group differed from the drug use and alcohol groups on the awareness stage: individuals with gambling problems were less likely to be aware of stigma than people with substance use or alcohol problems.

**Conclusion:** The internalization of stigma among individuals with gambling problems tends to work in a similar way as for those with alcohol or drug problems. The differences between the gambling group and the alcohol and other substance groups at the aware stage may suggest that public stigma with regard to any given addictive disorder may be a function of the type of addiction (substance versus behavioral).

## Introduction

One of the important changes in the Fifth Edition of the *Diagnostic and Statistical Manual of Mental Disorders* (DSM-5: American Psychiatric Association [APA], 2013) was to include Gambling Disorder under the section on Substance-Related and Addictive Disorders for the first time (Straussner, 2013). The decision to include gambling disorder in this section reflects the acknowledgment that this disorder is comorbid with substance use disorders, and is similar to them as regards certain symptom presentations, genetic liability, biological dysfunctions and treatment approaches (Hasin et al., 2013; Petry, 2015).

However, the similarities between these disorders may not be manifested in relation to public stigma; i.e., the prejudice and discrimination directed at a group by the population at large (Corrigan and Rao, 2012). Previous studies that compared public stigma across these addictive disorders (gambling vs. alcohol and other substances) suggest that people with alcohol or substance dependence are viewed by the public more negatively than pathological gamblers (Feldman and Crandall, 2007; Hing et al., 2016b). For example, Hing et al. (2016b) asked a sample of 2000 adult residents of Victoria, Australia to read five vignettes about recreational gambling, problem gambling, alcohol use disorder, schizophrenia, and a subclinical distress control. They found that problem gambling was less stigmatized than alcohol use. Similar results were reported by Feldman and Crandall (2007) in a study exploring which mental disorders are the most stigmatized or socially rejected. Based on a sample of 270 American university students who read case histories describing individuals with 40 mental disorders, they found that out of the addictive disorders, cocaine dependence was rated as the most stigmatized disorder (rated 6th), more than alcohol dependence (rated 10th) whereas pathological gambling was rated 13th. In contrast, one study also based on a student sample (this time249 Canadian university students) who rated vignettes describing males with five different health conditions found no differences between disordered gamblers and alcohol dependence in terms of desired social distance from these disorders (Horch and Hodgins, 2008). In Israel, where the current study was conducted, an earlier study indicated that mental health professionals perceived the issue of adolescent gambling as less severe than alcohol or drug use (Sansanwal et al., 2016).

However, public stigma does not stop there since it can also be internalized (Schomerus et al., 2014). Exposure to public stigma may lead to self-stigma – a process that integrates emotional and cognitive elements – which accrues when a person applies this internalized common negative public stigma to herself/himself (Corrigan et al., 2006). Once this process occurs, the individual may exhibit negative emotional reactions such as poor self-efficacy and diminished self-esteem (Corrigan and Rao, 2012). This process also impedes treatment-seeking and recovery among individuals with gambling (Hing et al., 2016a) and other substance use problems (Luoma et al., 2014). Hence, self-stigma is the harmful impact that results from internalizing prejudice (Corrigan et al., 2012).

Recently, the issue of self-stigma has attracted growing attention in the field of gambling research (Horch and Hodgins, 2015; Hing et al., 2016a). Using qualitative methods, Hing et al. (2016a) showed that problem gamblers have strong feelings of self-stigma. Horch and Hodgins (2015) reported that self-stigma was associated with increased shame and reduced self-esteem in individuals with a gambling disorder. These findings are in line with studies on individuals with substance use problems that have documented high levels of self-stigma (Luoma et al., 2007; Etesam et al., 2014), and found associations between self-stigma and internalized shame and reduced self-esteem (Rodrigues et al., 2013; Luoma et al., 2014). According to Donaldson et al. (2015), people with gambling problems may share characteristics with individuals with alcohol and substance abuse associated with the experience of stigma related to their condition. Specifically, they all have high rates of comorbidities and co-stigmas considered to be adaptive disorders, where stigma often acts as a barrier to treatment and affects treatment- seeking.

One of the key attempts to account for the cognitive and emotional process of self-stigma is the Progressive Model of Self-stigma, which emphasizes its developmental and multilevel processes (Corrigan et al., 2006, 2011). In this model, self-sigma consists four successive interrelated stages: awareness (aware), agreement (agree), application (apply) and harm. Each stage is the precondition for the next one, which is trickle-down in nature. Awareness is the first stage of this cascade of stigmatizing cognitions that denotes the person’s awareness of beliefs about mental illness in the culture in general. This stage actually represents the individual’s perception of public stigma (Schomerus et al., 2011). This stage may lead to agreement with the stigma, where an individual with a serious mental illness believes that the stereotype is true. Subsequently, the individual concurs that these stereotypes apply to him/herself, which finally leads to the experience of harm such as loss of self-esteem. The most harmful effects of self-stigma are thought to occur in the latter stages, when a person has internalized the stigma (Corrigan et al., 2012). For example, apply and harm (the last two stages) yielded significantly greater associations with self-esteem and the negative impact of hopelessness (Corrigan et al., 2011).

In practice, two assumptions are derived from this model, which are tested in two ways (Corrigan et al., 2011, 2012; Schomerus et al., 2011): the first is the trickle-down nature of the model which requires that self-stigma scores should be the highest in aware, decline progressively thereafter, and be the lowest for the last stage of harm. The second assumption leads to the prediction that cross-step correlations should be larger for steps representing proximal (e.g., aware-agree, agree-apply) than more distant stages (aware-apply, agree-harm, or aware-harm). Several studies have tested these assumptions. Although the progressive nature of the model was supported in several (Schomerus et al., 2011; Corrigan et al., 2012), others have only lent partial support to the trickle-down nature of the process (Rüsch et al., 2006; Corrigan et al., 2011). For example, in the Corrigan et al. (2012) study, the awareness stage was significantly higher than agreement, which was higher than values of apply and harm. However, no differences were found between apply and harm. The authors concluded that the stages are split into two sets between agree and apply.

With regard to individuals with addiction disorders, Schomerus et al. (2011) tested this model on 153 individuals with a diagnosis of alcohol dependence and showed that the stepwise process of self-stigmatization in this sample was similar to the pattern observed in people with other severe mental health illnesses. However, despite these accumulating findings, to the best of our knowledge, the process of self-stigmatization formation has not been empirically explored in individuals with a gambling disorder or compared to individuals with alcohol and other substance use disorders. Given the potential long-term consequences of self-stigma among those with gambling problems (Hing et al., 2016a) it is important to better understand the way self-stigma is formed. The current study was designed to probe the applicability of the Corrigan et al. (2006, 2012) progressive model of self-stigma to individuals with gambling problems. In addition, the inclusion of gambling disorder under the umbrella of substance-related and addictive disorders, and the similarities between these disorders raises the question of whether self-stigma forms and develops in the same way in these three disorders. The multi-dimensional nature of the progressive model can be used to explore this model as a whole and determine whether it unfolds in the same way among individuals with gambling, alcohol and other substance use problems. In addition, it can reveal potential differences in self-stigma between individuals with gambling problems and individuals with alcohol and substance use problems at each stage.

Based on a literature review, two hypotheses were tested: (1) the progressive nature of the self-stigma process among the individuals with gambling problems should emerge in the same way as among individuals with alcohol or other substance use problems. Namely, in all three groups (a) the mean scores in the early stages should be higher than the mean scores at later stages; and (b) the correlations between proximal stages should be larger than the correlations between distant stages; (2) Differences should only be found between individuals with gambling problems and individuals with alcohol and other substance use problems in relation to the awareness stage of the progressive model for self-stigma; i.e., individuals with gambling problems would be less likely to be aware of the stigma than individuals with alcohol and other substance use problems.

## Materials and Methods

### Sample and Procedure

This study is part of a wider longitudinal research project assessing a variety of psychological variables that predict dropping out from treatment in clinical populations of individuals with gambling, alcohol and other substance use problems (mainly heroin and cocaine). The criteria for inclusion in this study were: above age 18, residence in Israel for at least 10 years and the ability to read and write Hebrew sufficiently well to understand and fill in the questionnaires. For the purposes of this study, only individuals who met at least one item of the DSM-5 criteria related to gambling/alcohol or other substance use disorders were included in the analyses.

The sample was composed of 148 individuals who applied for treatment in out-patient rehabilitation centers for gambling, alcohol, other substances addictions in Israel. Of these, 37 individuals had gambling problems, 60 had alcohol use problems and 51 had other substance use problems. Two additional participants were excluded due to missing data. A research assistant was present at the rehabilitation treatment centers the day the subjects applied for treatment intake. After the individuals finished the intake procedure the research assistant asked them to take part in the study, and to read and sign the informed consent form. The subjects completed anonymous, confidential self-report measures, which were administered in the form of face-to-face interviews. The data were collected between 2015 and 2016. All study procedures were reviewed and approved by the Tel Aviv University Institutional Review Board and the Ministry of Welfare Review Boards. The study was conducted in accordance with the ethical standards of the American Psychological Association.

### Measures

**The Self-Stigma of Mental illness Short Form (SSMIS-SF)** was developed by Corrigan et al. (2012) to evaluate self-stigma among people with mental-health illnesses. It contains 20 items divided into four subscales representing awareness, agreement, application, and harm to self-esteem. Each stage is represented by five items; for example, “I think the public believes most people with mental illnesses are unpredictable” represents the awareness stage, whereas the item: “I currently respect myself less because I am unpredictable” represents harm to self. Agreement with each item is expressed on a nine-point scale ranging from 1 (*strongly disagree*) to 9 (*strongly agree*). Scale scores are determined for each of the subscales separately by summing only the five items for each subscale with the highest scores, which is considered to indicate greater endorsement of self-stigma for that factor. The scale was modified for the purposes of this study to refer to gambling, alcohol and other substance use addictions by changing the term “mental illness” to gambling, alcohol or other substance use addictions as appropriate. The reliability of the subscales has been tested on different samples (Corrigan et al., 2012). Awareness ranges from α = 0.73–0.87, agreement (α = 0.72–0.79), application (α = 0.22–0.74), and harm to self (0.76–0.82). In the current study, the reliabilities were α = 0.72 for awareness, α = 0.68 for agreement, α = 0.66 for application, and α = 0.82 for harm. All four self-stigma sub-scales distributed normally: awareness (skewness = -0.385 and kurtosis = -0.757); agreement (skewness = -0.034 and kurtosis = -0.416); application (skewness = 0.592 and kurtosis = -0.518), and harm (skewness = 0.768 and kurtosis = -0.446).

**The severity of the addiction disorders** was assessed by DSM-5 diagnostic criteria for gambling, alcohol and other substance addictions separately. On the DSM-5, gambling disorder is assessed by 9 criteria, and alcohol and other substance use disorders are assessed by 11 criteria each. The participants were asked to think about the previous 12 months and to choose one answer for each criterion. As stipulated in the DSM-5 GD guidelines, individuals with a score of 4 or above were considered disordered gamblers and were sub-classified as having a mild (met 4–5 criteria), moderate (met 6–7 criteria) or severe (met 8–9 criteria) gambling disorder. For alcohol and other substance use disorders, individuals who scored 2 or above were considered disordered alcoholics or as having a substance disorder and were sub-classified as having mild (met 2–3 criteria), moderate (met 4–5 criteria), or severe (met 6 or more criteria) alcohol or other substance use disorders. To compare the severity levels of the three addiction problems a four-level scale (no severity/mild/moderate/severe) corresponding to the DSM-5 was used. In addition, socio-demographic information was collected for gender, level of education and age.

### Statistical Analyses

Analyses were carried out with SPSS24 and AMOS 24 for Windows. First, the data were scanned to identify missing values. Only eight participants did not fill in all the items (between 1–5 missing items) on the self-stigma questionnaire. The missing values were replaced by the series mean.

A confirmatory factor analysis was used to test whether the factorial structure of the SSMIS-SF developed for mental illness was also applicable to people with gambling, alcohol and other substance use problems. After omitting item numbers 1 and 3 on the awareness subscale, items number 1 and 3 on the agreement subscale, item number 3 on the application subscale, and item number 3 on the harm scale, the stigma scale in the current sample showed good fit indices [χ^2^(60) = 78.324, χ^2^/*df* = 1.305, CFI = 0.978, TLI = 0.967, and RMSEA = 0.046]. Values greater than 0.90 for CFI and TLI and values ranging from 0.06 to 0.08 for RMSEA are generally deemed acceptable (Browne and Cudeck, 1993; Hu and Bentler, 1999; Kline, 2011).

Descriptive statistics were calculated to characterize the groups in terms of gender, age, educational level and addiction severity. In addition, ANOVA, Kruskal–Wallis and Fisher’s exact tests were conducted to identify demographic differences between the groups. Since significant differences in gender, age, education, and addiction severity were found, these variables were controlled for in the subsequent analyses. Relationships between demographic variables and self-stigma measures were examined using MANOVA and Pearson correlations. The analyses of the progressive model assumptions followed methodology used in previous studies (Corrigan et al., 2011, 2012; Schomerus et al., 2011). The relationships between self-stigma stages were evaluated by partial correlations. The differences between stage scores, and between-group differences were evaluated by implementing repeated measures ANCOVA and one way MANCOVA.

## Results

### Sample Description

The distributions of the demographic variables in the three groups are presented in **Table 1**. Females were a minority in all three groups, but gender proportions were group-dependent: 15% of the alcohol group, 11.8% of the other substance use group and only 2.7% of the gambling group were women. Significant age difference was found [*F*(2,147) = 5.86, *p* < 0.01, η^2^ = 0.074]; namely, participants with gambling problems were younger than participants with alcohol problems (Bonferroni *post hoc* test, *p* < 0.005), whereas the age of participants with substance use problems did not differ from either group. Participants with other substance use problems were less educated than participants with alcohol or gambling problems [χ^2^(2) = 17.59, *p* < 0.001, η^2^ = 0.120]. Significant differences were found in terms of addiction severity [χ^2^(2) = 15.25, *p* < 0.001, η^2^ = 0.104]. The overall addiction severity was significantly lower among individuals with gambling problems than in individuals with alcohol and other substance use problems (the difference between individuals with alcohol and other substance use problems was not significant). MANOVA and Pearson correlations analyses revealed no significant relationships between demographic variables and self-stigma measures.

**Table 1:** Demographic data.

	Gambling (*N* = 37)	Alcohol (*N* = 60)	Substances (*N* = 51)	Group differences	Effect size
Gender – female *N* (%)	1 (2.7%)	9 (15%)	6 (11.8%)	FET^∗∗^	*V* = 0.17
Age – *M* (*SD*)	35.00 (9.28)	43.31 (12.56)	39.60 (12.11)	*F*(2,147) = 5.86^∗∗^	η^2^ = 0.074
Education level mean rank	80.81	85.42	57.08	χ^2^(2) = 17.59^∗∗∗^	η^2^ = 0.120
Up to 8 years *N* (%)	2 (5.4)	4 (6.7)	17 (33.3)		
Up to 12 years *N* (%)	27 (73)	38 (63.3)	28 (54.9)		
Non-academic *N* (%)	3 (8.1)	7 (11.7)	6 (11.8)		
Academic *N* (%)	5 (13.5)	11 (18.3)	–		
Addiction severity – mean rank	56.35	77.73	83.86	χ^2^(2) = 15.25^∗∗∗^	η^2^ = 0.104
Severe *N* (%)	16 (43.2)	47 (78.3)	44 (86.3)		
Moderate *N* (%)	19 (51.4)	4 (6.7)	2 (3.9)		
Mild *N* (%)	1 (2.7)	7 (11.7)	3 (5.9)		
No severity (Met only 1 DSM item) *N* (%)	1 (2.7)	2 (3.3)	2 (3.9)		

*^∗∗^*p* < 0.01, ^∗∗∗^*p* < 0.001*.

### The Progressive Model of Self-stigma in Individuals with Gambling, Alcohol and other Substance Use Problems

The first hypothesis examined whether the progressive model of self-stigma could apply to individuals with gambling problems as it has been shown to apply to those with alcohol and substance use problems. Thus, the assumptions of the progressive model were tested for each group separately.

**FIGURE 1:**
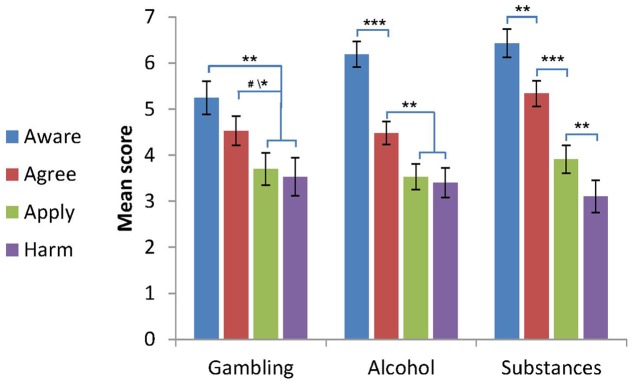
Self-stigma scores by group (M ± SE, gender, age, education level and addiction severity were controlled for). Notations: The figure shows the progressive patterns of stage scores for each group. Gambling: aware = agree > apply = harm. Alcohol: aware > agree > apply = harm. Other substances: aware > agree > apply > harm. ^∗^*p* < 0.05, ^∗∗^*p* < 0.01, ^∗∗∗^*p* < 0.001, ^#^*p* < 0.06, compared to the signed stages in each group (for the gambling group, *p* < 0.06 for the agree-apply difference, *p* < 0.05 for the agree-harm difference).

The first assumption of the progressive model has to do with the putative differences between stage scores. According to the model, early stage scores should be higher than later stage scores (Corrigan et al., 2011). This hypothesis was tested using a repeated measures ANCOVA, with stage (aware/agree/apply/harm) as a within-subjects independent factor, group (gambling/alcohol/substances) as a between-subjects independent factor, and gender, age, education and severity as the control variables. A significant stage^∗^group interaction was found [*F*(6,423) = 2.83, *p* = 0.01, η^2^ = 0.039]. Overall, as can be seen in **Figure 1**, the stages scores in each group were in line with the progressive model’s first assumption; namely, the mean scores in the early stages were higher than the mean scores at later stages. However, Bonferroni *post hoc* tests for the interaction effect showed a slightly different pattern in each group: as can be seen in **Figure 1**, in individuals with gambling problems, the aware score was higher than the apply and harm scores (*p* < 0.005), and the apply score tended to be higher than the agree score (*p* < 0.06) and was higher than the harm score (*p* = 0.05). No significant differences were found between the aware and agree scores, or between apply and harm scores. In individuals with other substance problems, the differences between all the stages were significant (*p* < 0.01): the aware score was the highest, the agree score was lower than the aware, the apply score was lower than the agree, and the harm score was the lowest. In individuals with alcohol problems, the pattern was similar except for the absence of a significant difference between apply and harm scores. Thus overall, the first two stage scores were higher than the last two stage score in all groups, which is consistent with the assumptions the progressive model. This effect is presented in **Figure 1**.

The second assumption derived from the self-stigma progressive model states that proximal stages in the hierarchy are more highly correlated than relatively distant stages. Partial correlations representing the relationships between scales (after controlling for age, gender, education and addiction severity) are presented in **Table 2**. As shown in **Table 2**, this hypothesis was partially supported. For example, proximal correlations were high for apply/harm (0.63/0.85/0.71 for the gambling, alcohol and other substances groups, respectively) compared to more distant relationships between aware and harm (0.26/0.27/0.38). In order to make sense of all these correlation coefficients, we followed the Corrigan et al. (2012) method and calculated the mean of the correlation coefficients for each proximity level in each group (after a Fisher r to z transformation). The results were as expected: in all three groups, the correlations were the highest for the proximal relationships, lower for the intervening and the lowest for the most distant (Gambling: 0.56/0.54/0.26; Alcohol: 0.68/0.39/0.27; Substances: 0.53/0.35/0.38). In fact, in individuals with other substance use problems, the second and third level correlations were close, and both were lower than first level correlations. Thus, the progressive nature of relationships between the self-stigma stages was confirmed in all three groups.

**Table 2:** Partial correlations between self-stigma scales by group.

	Gambling (*N* = 37)			Alcohol (*N* = 60)			Substances (*N* = 51)		
	Aware	Agree	Apply	Aware	Agree	Apply	Aware	Agree	Apply
Aware	-			-			-		
Agree	0.45^∗∗^	-		0.44^∗∗∗^	-		0.43^∗∗^	-	
Apply	0.31^#^	0.58^∗∗∗^	-	0.26	0.60^∗∗∗^	-	0.36^∗^	0.39^∗∗^	-
Harm	0.26	0.71^∗∗∗^	0.63^∗∗∗^	0.27^∗^	0.51^∗∗∗^	0.85^∗∗∗^	0.38^∗∗^	0.34^∗^	0.71^∗∗∗^

*^∗^*p* < 0.05, ^∗∗^*p* < 0.01, ^∗∗∗^*p* ≤ 0.001, ^#^*p* < 0.06 *(gender, age, education level, and addiction severity were controlled for)**.

### Differences between Groups in Self-stigma Stages

The second hypothesis related to potential differences between individuals with gambling problems and individuals with alcohol and other substance use problems in the self-stigma stage scores. This hypothesis was tested using a one-way MANCOVA, with four stages scores (aware/agree/apply/harm) as dependent variables, group (alcohol/substances/gambling) as an independent factor, and gender, age, education and severity as the control variables.

The multivariate effect was significant [*F*(8,272) = 2.40, *p* > 0.05, η^2^ = 0.066]. Univariate analyses results, together with self-stigma scores in each group, are presented in **Table 3**. As can be seen in the table, a significant effect was found only in the awareness score [*F*(2,139) = 3.71, *p* < 0.01, η^2^ = 0.051]. Bonferroni *post hoc* tests showed that the aware score among individuals with gambling problems was lower compared to individuals with other substances use problems (*p* < 0.05) and tended to be lower also compared to individuals with alcohol problems (*p* < 0.06). No between-group difference was found considering the other stages scores.

**Table 3:** Self-stigma scores by group.

	Gambling (*N* = 37)		Alcohol (*N* = 60)		Substances (*N* = 51)		Group differences	Effect size
	*M*	*SE*	*M*	*SE*	*M*	*SE*	*F*(2,139)	η^2^
Aware	5.25	0.36	6.19^#^	0.28	6.43^∗^	0.31	3.71, *p* < 0.05	0.051
Agree	4.53	0.32	4.48	0.25	5.34	0.28	2.61, *p* > 0.05	0.036
Apply	3.70	0.35	3.53	0.28	3.91	0.30	0.23, *p* > 0.05	0.003
Harm	3.53	0.41	3.40	0.32	3.10	0.35	0.47, *p* > 0.05	0.007

*^∗^*p* < 0.05, ^#^*p* < 0.06, both compared to gambling group *(gender, age, education level and addiction severity were controlled for)**.

## Discussion

This study examined the applicability of the progressive model of self-stigma to individuals with gambling problems, and probed whether its assumptions applied in the same way as among individuals with alcohol and other substance use problems who had sought treatment at rehab centers in Israel. It also compared the three groups with respect to each of the four phases in the model. The findings partially confirmed the model’s first assumption (its trickle-down nature) and came close to confirming its second assumption (that cross scale correlation coefficients between proximal stages would be larger than distal ones), with only minor differences between the groups. With regard to the first assumption, which posits that mean scores in early stages should be higher than the following ones, in all three groups differences were found between the first two stages of the model – aware and agree versus the other two stages – apply and harm (in the gambling group this difference came close to significance). However, in subjects with an alcohol or substances use problem there was a significant difference between the aware and agree stages, and in the group of individuals with substance use problems there was also a significant difference between the apply and harm stages. The second assumption that cross-scale correlation coefficients between proximal stages would be larger than with distal ones was fully borne out in the gambling and alcohol groups, but only partly in the substance use group, where the cross-scale correlation coefficients between proximal stages were indeed larger than in the distal stages, but the correlation coefficients between the medial-distant stages were similar to the distal. In line with the second hypothesis, which compared the groups with regard to each stage individually, the analyses revealed differences for the aware stage between participants in the gambling group and those in the alcohol and other substance use groups (with the alcohol group the difference was marginally significant). Specifically, participants in the gambling groups had a lower awareness of stigma than those with alcohol and other substance use problems. There were no significant differences between the groups for the three later stages of the model; namely, agree, apply and harm.

These findings highlight the similarities and differences in self-stigma development in individuals with gambling problems as compared to those with alcohol and substance use problems. These differences and similarities held true for each of the constituent stages of this process, and with regard to the progression of self-stigma as a whole.

The differences between the gambling group and the alcohol and other substance use groups regarding the aware stage highlight this difference. The aware stage is a reflection of the public’s stigma toward a given behavior, as perceived by the members of the stigmatized group (Schomerus, 2014). The findings with regards to this stage that participants with gambling problems scored lower than those with substance use and alcohol problems suggests that (in Israel, at least) these disorders can be divided into two levels of severity of public stigma. This finding echoes the results in Feldman and Crandall (2007) in the United States showing that people were less inclined to avoid the company of pathological gamblers than those with an alcohol dependence, and were most inclined to avoid people with a cocaine dependence. This finding may be accounted for by the idea that in contrast to substance addictions, in gambling – which is a behavioral addiction – the damage to physical appearance is not as prominent, is much easier to conceal (Horch and Hodgins, 2008; Donaldson et al., 2015), and hence may attract lower levels of public stigma. Therefore, public stigma with regard to any given addictive disorder may be a function of the type of addiction (substance versus behavioral).

As previously noted, the first hypothesis concerned the applicability of the progressive model to the three groups and was tested to acquire a deeper understanding of the stages of development of self-stigma in individuals with gambling, alcohol and substance use problems. The findings revealed that the principles of the model were broadly substantiated in all three groups, with only minor differences that may have been due to the size of the groups. In all three groups there was a difference between first two stages (aware and agree) and the latter stages (apply and harm). This finding is in line with results reported by Rüsch et al. (2006) and Corrigan et al. (2011), which also found differences between the first two stages (which concern the cognitive aspect of the development of stigma) and the latter two stages, which tend to relate to the practical process of internalization and the harm caused to the individual from the stigma. The larger associations between the proximal stages, and the weaker associations with the distal stages (fully in the case of the gambling and alcohol groups, and partially in the substance group) substantiate the model for the three groups, inasmuch as the internalization of the stigma was quite similar in all the addiction disorders. Thus, the findings of this study on self-stigma tend to support the similarities found in the literature in terms of the characteristics of behavioral and substance use disorders (Hasin et al., 2013). It is important to note that a larger sample, particularly with more subjects with gambling problems, could very well have led to a more decisive corroboration of the model, including in relation to this group.

Although the link between self-stigma and socio-demographic variables was not the main purpose of this study, it is important to note that no associations were found between the self-stigma subscales and gender, age, or level of education. These findings are in line with a study conducted by Brown et al. (2015) which probed the potential associations between self-stigma and demographic and previous treatment variables among 120 individuals residing in a Midwestern U.S. state substance use facility. The authors concluded that demographic variables, including gender, do not seem to be particularly relevant with regard to self-stigma. However, given the notion that women with gambling problems bear a dual stigma as a result of having both gambling problems and because of their failure to meet certain social gender expectations (Lesieur and Blume, 1991; Brown and Coventry, 1997) the findings – both in this study and in previous work – are surprising. More studies should be conducted on women and men separately using qualitative and quantitative methods.

The current study found no associations between addiction severity and the self-stigma sub-scales. Given previous findings which have found a relationship between substance use diagnosis and the self-devaluation and fear of enacted stigma scales (Brown et al., 2015), and a link between the apply and harm subscales of the SSMIS-SF and severity of drinking problems in 153 patients hospitalized for alcohol detoxification (Schomerus et al., 2011) more studies should be conducted to clarify this issue.

Understanding self-stigma and its development is crucial to reducing its adverse effects on the individual, at all stages of treatment – seeking treatment, treatment itself and recovery. The findings show that despite the differences between the groups in the first stage of the model, there was no difference between the groups for the agree, apply and harm stages, and the groups fell broadly in line with the model’s assumptions in general, as was shown by the divisions between the early and later stages. Hence, these differences and similarities between groups should be reflected in prevention programs as well. In terms of the process of self-stigma development as a whole, the same practices should be implemented for all individuals who suffer from self-stigma stemming from their addictive disorders (whether behavioral or substance related). However, the differences between the groups in the aware stage emphasize the need to develop tailored interventions programs that take different public attitudes into account.

This study also has a number of limitations. Most studies have argued that high stigma is a major deterrent to seeking treatment for people with gambling problems (Rockloff and Schofield, 2004; Gainsbury et al., 2014; Hing et al., 2016a), as well as for those with alcohol and other substance problems. Since the participants in this study all actively sought treatment, it is possible that they experienced lower levels of self-stigma than individuals who avoid doing so. In addition, this study was based on self-reports with no cross-referencing to other sources, and on a relatively small sample. Further studies should be conducted with larger numbers of participants – both those who have sought treatment and those who have not. Despite these limitations, this study contributes to the body of knowledge on the stages in which self-stigma develops among individuals with gambling problems, and is the first study to compare the assumptions of the progressive model on a clinical sample of individuals with alcohol and other substance use problems to individuals with gambling problems. The findings of this study should thus pave the way for further studies in this field.

## Author Contributions

BG-F wrote the manuscript and was involved in the statistical analyses. TR was involved in solidifying the main research questions and was responsible for some aspects of the methodological part of the manuscript and for the data collection. The authors discussed the research findings and their implications together. Overall, BG-F and TR contributed significantly to this study.

## Conflict of Interest Statement

The authors declare that the research was conducted in the absence of any commercial or financial relationships that could be construed as a potential conflict of interest.
